# Validation of the AKIN criteria definition using high-resolution ICU data from the MIMIC-II database

**DOI:** 10.1186/cc9525

**Published:** 2011-03-11

**Authors:** T Mandelbaum, DJ Scott, J Lee, RG Mark, MD Howell, A Malhotra, D Talmor

**Affiliations:** 1 Beth Israel Deaconess Medical Center and Harvard Medical School, Boston, MA, USA; 2 MIT-HST, Cambridge, MA, USA; 3 Brigham and Women's Hospital and Harvard Medical School, Boston, MA, USA

## Introduction

Recently the Acute Kidney Injury (AKI) Network proposed criteria for the definition of AKI in the critically ill. The minimum hourly urine output rate used to define oliguria (< 0.5 ml/kg/hour) is based exclusively on clinical experience and animal models, not on clinical investigation. Moreover, the minimum duration of oliguria (6 hours) is based on clinical experience and was never experimentally determined. We used a massive database of ICU patients (MIMIC) to continuously vary the observation period and threshold of urine output measurements to determine optimal AKI definitions for improved in-hospital mortality prediction.

## Methods

After excluding end-stage renal disease, 14,536 adult patients were included. Various AKI thresholds corresponding to different observation periods and urine output measurement thresholds were analyzed using a multivariate logistic regression model for each choice of thresholds. A total of 470 regression models were plotted. We controlled for sex, age, SOFA and co-morbidities (ICD-9 codes). To visualize dependence of adjusted mortality rate and mortality predictive power on AKI definition, we generated 3 D and contour plots.

## Results

**Figure 1:**
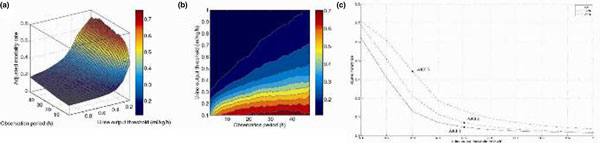
**(a), (b) Urine output mortality plot**. **(c) **Cross-section at 6, 12, 24 hours.

The UO versus mortality plot demonstrates that when UO <0.5 the mortality rate increases rapidly as urine output decreases. Mortality increases sharply for observation periods up to 5 hours and then the rate of increase is reduced until a plateau is reached at approximately 24 hours. Cross-sections at 6, 12 and 24 hours of the UO mortality plot shows that the mortality rate of AKI 1 and AKI 2 are similar but differ significantly from AKI 3. See Figure 1.

## Conclusions

The current AKIN recommendation that uses a urine output of 0.5 ml/kg/hour is valid. Since AKIN's stages 1 and 2 were found to exhibit similar mortality rates, we propose a reduction in the AKI 2 threshold to 0.4 ml/kg/hour to better delineate among the three stages. We demonstrated that the mortality rate increases sharply during the first 5 hours of oliguria. Hence, the current used observation period (6 hours) seems to be valid.

